# Developing and Tailoring a Nutrition Intervention for Dementia Risk Reduction

**DOI:** 10.1002/alz.090938

**Published:** 2025-01-03

**Authors:** Julia L Sheffler

**Affiliations:** ^1^ Florida State University College of Medicine, Tallahassee, FL USA

## Abstract

**Background:**

Nutrition and other lifestyle interventions hold promise for reducing dementia risk; however, significant barriers remain in translating these programs and recommendations to individuals at the greatest risk. We discuss application of the NIH Obesity‐Related Behavioral Intervention Trials (ORBIT) to the development and tailoring of a nutrition program for high‐risk older adults.

**Method:**

A mixed methods approach was used to evaluate factors that contribute to adherence and engagement with the nutrition program. Quantitative and qualitative data were drawn from two early phase clinical trials. Additionally, focus group data is reported to supplement the trial data.

**Result:**

Program‐specific factors (e.g., group cohesion, delivery methods, quality of materials, and support for change) and personal factors (e.g., supportive family, health conditions, food‐related habits/experience) were identified as potential contributors to adherence and engagement. Further, focus groups provided specific feedback and recommendations about updates that can enhance adherence and engagement with the nutrition program.

**Conclusion:**

Intervention development and adaptation is an iterative process that can be enhanced through mixed methods approaches that provide qualitative context for quantitative trial results.

